# P04.13. Population-based case-control study of Chinese herbal products containing ephedra and cardiovascular disease risk

**DOI:** 10.1186/1472-6882-12-S1-P283

**Published:** 2012-06-12

**Authors:** Y Lin, N Hu, L Chang, M Hou

**Affiliations:** 1 China Medical University, Taichung, Taiwan; 2 Department of Information Management, National Formosa University, Yunlin, Taiwan; 3 Department of Chinese Medicine, Changhua Christian Hospital, Changhua, Taiwan

## Purpose

After reviewing some adverse events, such as cardiovascular disease, regarding the use of ephedra-containing dietary supplements, the Food and Drug Administration banned the sale of ephedra products on April 12, 2004. However, according to standard prescriptions recommended by the Committee on Chinese Medicine and Pharmacy in Taiwan, ephedra is still a popular ingredient of many Chinese herbal formulas. This paper examined the association between prescribed ephedra-containing Chinese herbal products (CHP) and cardiovascular disease by using the population-based database in Taiwan.

## Methods

All patients newly diagnosed with cardiovascular disease (CVD) from 2006 to 2007 as case subjects, and a random sample of the entire insured population from 1997 to 2007 excluding patients with CVD, including myocardial infarction, stroke, arrhythmias, or cardiac sudden death, as control subjects, were selected from the National Health Insurance reimbursement database. Subjects with incomplete data or any hyperlipidemia-related diagnosis were also excluded. For the association between prescribed ephedra-containing CHP and the occurrence of CVD, we used multivariable logistic regression models to estimate odds ratios and 95% confidence intervals. Models were adjusted for age, sex, residence, prescription of acetazolamide and/or labetalol, and cumulative doses of prescribed ephedra-containing CHP.

## Results

There were 1,120 case subjects and 41,409 control subjects in the final analysis. There was a significant reduction of CVD development for consuming ephedra-related CHP (OR=0.183, p<0.001). Only three items (Xiao Xu Ming Shang OR=2.212, p<0.001; Gui Qi Shao Yao Zhi Mu Shang OR=1.701, p<0.001; She Gan Ma Huang Shang OR=1.441, p=0.006) out of a total of 24 ephedra-related CHP were associated with the risk of CVD development. No statistically linear dose-response relationship was observed with the prescribed dose of ephedra.

## Conclusion

Consumption of ephedra-containing CHP does not increase the occurrence of CVD.

